# [Corrigendum] Aloperine activates the Nrf2‑ARE pathway when ameliorating early brain injury in a subarachnoid hemorrhage model

**DOI:** 10.3892/etm.2024.12552

**Published:** 2024-04-25

**Authors:** Shibin Song, Yimin Chen, Feng Han, Minghao Dong, Xin Xiang, Jianmei Sui, Yuming Li, Hua Yang, Jian Liu

Exp Ther Med 15:3847–3855, 2018; DOI: 10.3892/etm.2018.5896

Following the publication of the above article, an interested reader drew to the authors’ attention that the data panels for Fig. 3B and C on p. 3851 (representing the SAH and the SAH + vehicle groups, respectively) appeared to be overlapping, such that the data may have been derived from the same original source where they were intended to have shown the results from differently performed experiments. The authors have re-examined their original data, and realized that the data in this figure had inadvertently been assembled incorrectly.

The revised version of Fig. 3, now incorporating data for Fig. 3B and C from one of the alternative experiments, is shown opposite. Note that the error made in assembling this figure did not have an impact on either the results or the conclusions reported in the paper. All the authors agree with the publication of this corrigendum, and are grateful to the Editor of *Experimental and Therapeutic Medicine* for allowing them the opportunity to publish this. Moreover, they apologize to the readership for any inconvenience caused.

## Figures and Tables

**Figure 3:**
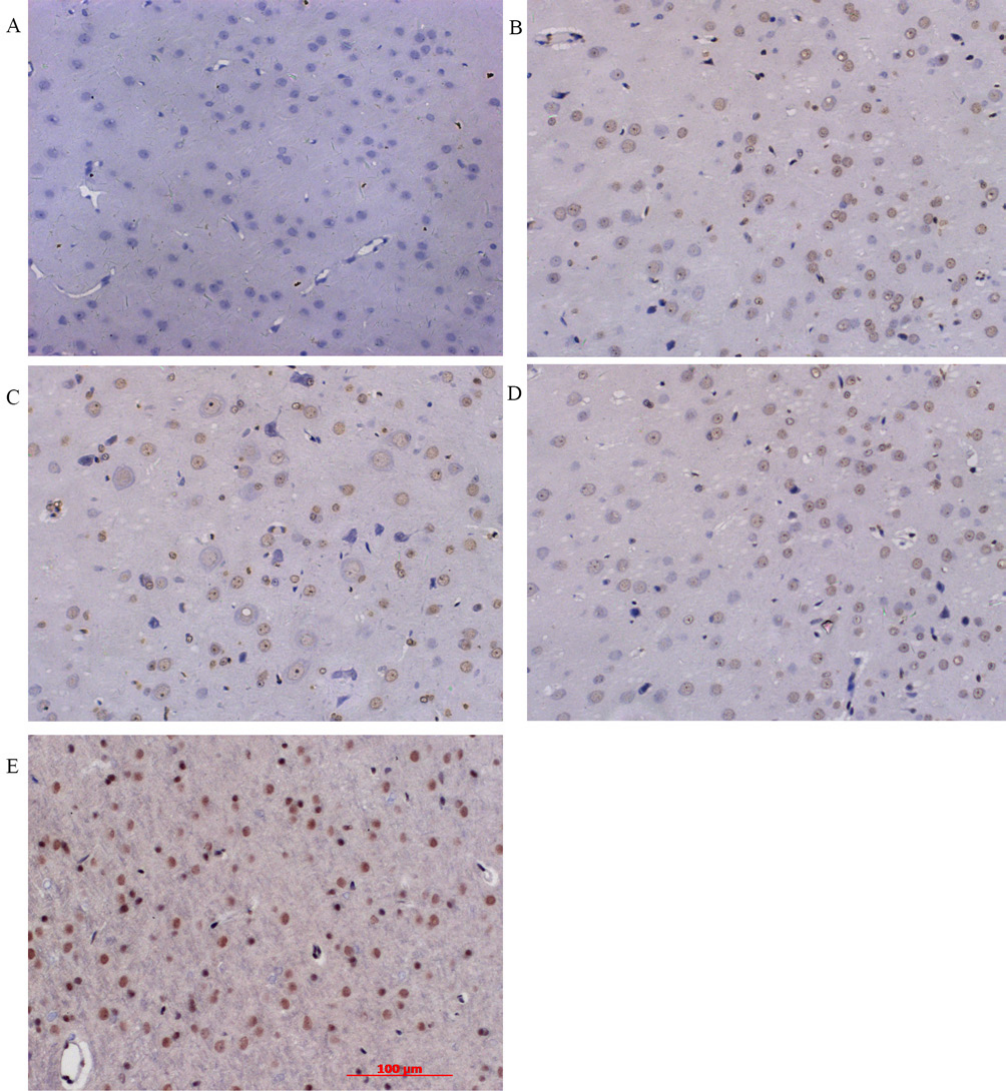
Immunohistochemical analysis of HO-1 in cortex. (A) sham group; (B) SAH group; (C) SAH + vehicle group; (D) SAH + low concentration ALO group; (E) SAH + high concentration ALO group (scale bar, 100 µm). HO-1, heme oxygenase-1; ALO, aloperine; SAH, subarachnoid hemorrhage.

